# Editorial: Molecular Mechanisms for Reprogramming Hippocampal Development and Function by Early-Life Stress

**DOI:** 10.3389/fnmol.2016.00006

**Published:** 2016-02-01

**Authors:** Xiao-Dong Wang, Mathias V. Schmidt

**Affiliations:** ^1^Key Laboratory of Medical Neurobiology of Ministry of Health of China, Zhejiang Province Key Laboratory of Neurobiology, Department of Neurobiology, Zhejiang University School of MedicineHangzhou, China; ^2^Department of Stress Neurobiology and Neurogenetics, Max Planck Institute of PsychiatryMunich, Germany

**Keywords:** early-life stress, hippocampus, development, plasticity, molecular mechanism

The hippocampal formation is both a key component of the medial temporal lobe crucial for declarative memory and a main target of stress mediators (e.g., glucocorticoids and neuropeptides) and stress-related molecules (e.g., nutritional factors and cytokines). During the first weeks of life, the hippocampus significantly increases in volume (Zhang et al., 2005) and several critical developmental processes coincide: generation of new neurons, outgrowth of neurites, formation of synaptic contacts, and establishment of neuronal circuits (Khalaf-Nazzal and Francis, 2013). Although the neonatal hypothalamic-pituitary-adrenal (HPA) axis is relatively hyporesponsive to environmental challenges, age-appropriate stressors can activate stress response, which in turn alters hippocampal development and increases the risk to develop neuropsychiatric disorders later in life, dependent on adult life conditions, and genetic predispositions (for recent reviews, see Lucassen et al., 2013; Tost et al., 2015; Bick and Nelson, 2016; Chen and Baram, 2016). As many neuropsychiatric disorders, such as schizophrenia and anxiety disorders, have developmental origins (Gross and Hen, 2004; Howes and Murray, 2014), dissecting the molecular mechanisms mediating the potentially detrimental consequences of early-life stress will provide insight into the pathophysiology and intervention of these disorders.

Most studies so far focus on the mechanisms of the long-term impact of early-life stress on hippocampal plasticity in adolescence/adulthood, which are of clinical relevance. In comparison, molecular mechanisms on how stress shapes the developing hippocampus have received attention only recently (Gross et al., 2012; Wei et al., 2012, 2015; Suri et al., 2013; Liao et al., 2014). We therefore initiated this research topic to sum up recent findings with an emphasis on both the dynamic effects of early-life stress on hippocampal structure and function at different life stages and the immediate effects of stress on hippocampal development.

Firstly, Huang provides an overview on the molecular and cellular alterations that modulate the effects of prenatal or postnatal stress on hippocampal development, and discusses how epigenetic modifications underlie the programming effects of early-life stress and contribute to the pathogenesis of epilepsy (Huang). In the dentate gyrus (DG) of the hippocampal formation, new neurons are continuously generated and selectively integrated to local circuits throughout the lifespan. Unlike the adult DG where most granule cells are mature and settled in place, during the first postnatal week the infrapyramidal blade of DG is yet to be formed and a majority of neurons are still immature. Exposure to severe stressors may thus perturb various aspects of neonatal and adult hippocampal neurogenesis and evoke lasting behavioral consequences. Lajud and Torner describe the key processes of neonatal DG development and review the short-term, intermediate and lasting effects of early-life stress on hippocampal neurogenesis (Lajud and Torner). Koehl further discusses the interaction between environmental factors (including early-life stress) and genetic background in shaping adult hippocampal neurogenesis and propose a conceptual framework for identifying genes that confer stress resilience or vulnerability (Koehl).

Early-life adversities are also manifested by malnutrition or infection. The disruption of maternal care inevitably alters the levels of nutritional and inflammatory factors in the offspring, which may modulate the influences of early stress. Hoeijmakers and colleagues present a comprehensive update on the intricate interplay among these essential elements of early-life environment and discuss their synergistic effects in shaping hippocampal structure and cognition, with a specific focus on adult neurogenesis (Hoeijmakers et al.). Moreover, Lardner summarizes our current understanding on the involvement of vitamin D, a vital nutrient with pleiotropic effects that may be insufficiently available under early-life stressful situations, in hippocampal development (Lardner).

Neurotrophins, especially brain-derived neurotrophic factor (BDNF), regulate neural circuit formation and activity-dependent synaptic plasticity via Trk receptors. Daskalakis and colleagues address the cross-talk between glucocorticoids and BDNF-TrkB signaling in early stress-induced hippocampal maldevelopment and behavioral deficits (Daskalakis et al.). In the research report by Wang and colleagues, BDNF protein level is examined in the hippocampus, medial prefrontal cortex and nucleus accumbens at different time points after neonatal maternal separation, and sex difference is further compared and discussed (Wang et al.). These two articles highlight the modulatory role of BDNF in early postnatal stress-programmed hippocampal development.

The serotonin (or 5-hydroxytriptamine, 5-HT) system is a main molecular target for the intervention of depression and anxiety and implicated in the acute stress response. In another highlighted research article, Bravo and colleagues evaluate the mRNA levels of two key components of the serotonin system, 5-HT_1A_ receptor and serotonin transporter (SERT), in adult rats with or without a history of neonatal maternal separation, and find that early-life stress alters 5-HT_1A_ and SERT mRNA levels in the amygdala and dorsal raphe nucleus, but not the hippocampus (Bravo et al.). These alterations may underlie the susceptibility of early-life stressed individuals to affective or anxiety disorders.

For altricial animals such as mice and rats, somatosensory input from the skin/whisker provides a major information source for representation of early-life environment. Erratic maternal care and/or peer interaction may thus result in abnormal experience-dependent synaptic plasticity and reshape the development of neocortex and hippocampus. Takatsuru and Koibuchi review how early-life stress disrupts the structure and activity of the somatosensory cortex and suggest the involvement of glucocorticoids, glutamate, and microglia in stress-induced somatosensory alterations (Takatsuru and Koibuchi).

Taken together, this research topic summarizes recent progress on the mechanisms of the effects of early postnatal stress on hippocampal development, and underlines the interactions of various factors in programming hippocampal plasticity. Meanwhile, many more interesting questions and new challenges emerge, some of which are sketched below.

1. **Dynamics**. Neural development and plasticity are highly dynamic, so are the influences of early-life stress. It is important to explore how stress dynamically modulates the levels and activity of stress-related molecules, and how these molecular events affect the dynamics of neuronal structure (e.g., formation and elimination of dendritic spines) and activity at different life stages.
2. **Interactions**. Future studies need to balance between addressing the complex interactions (e.g., between the timing and features of the stressor and concomitant critical developmental events; between genetic makeup and environmental elements; sex differences; etc.) and maintaining a manageable experimental design.
3. **Pathways**. Our understanding on the mechanisms of early-life stress may benefit from studies extending from dissecting the molecular pathways to mapping anatomical (i.e., neural circuits) and functional (i.e., network activity) pathways.
4. **Adaptation**. While early-life adversity is undoubtedly a major risk factor for adult pathologies, not all alterations resulting from early-life stress may be detrimental. Mounting evidence suggests that at least some of the molecular, structural or functional consequences of early-life stress exposure are adaptive and may increase individual resilience to similar challenges later on. Future studies will therefore also address how the observed alterations affect vulnerability or resilience to additional challenges in adulthood.

In short, we hope this collection will provide new perspectives and stimulate studies on the molecular mechanisms of early-life stress and brain development.

## Author contributions

XW and MS wrote the manuscript.

### Conflict of interest statement

The authors declare that the research was conducted in the absence of any commercial or financial relationships that could be construed as a potential conflict of interest.
